# Intradural Chordoma Mimicking an Epidermoid Cyst on Imaging

**DOI:** 10.7759/cureus.40610

**Published:** 2023-06-18

**Authors:** Noriko Ito, Hiroyuki Fujii, Rintaro Kuroda, Mitsuru Matsuki, Harushi Mori

**Affiliations:** 1 Radiology, Jichi Medical University, School of Medicine, Tochigi, JPN; 2 Neurological Surgery, Jichi Medical University, School of Medicine, Tochigi, JPN; 3 Radiology, Jichi Children's Medical Center Tochigi, Tochigi, JPN

**Keywords:** magnetic resonance imaging, mri, epidermoid cyst, intradural chordoma, chordoma

## Abstract

Chordomas are rare, locally aggressive, primary bone tumors derived from primitive notochord remnants. They almost always arise within the axial skeleton, particularly in the skull base and the sacrococcygeal region. They usually present as extradural tumors, but rarely, they present as entirely intradural tumors. This report describes a case of intradural chordoma that mimicked an epidermoid cyst. A 72-year-old woman was incidentally found to have a prepontine extra-axial mass on magnetic resonance imaging. The mass gradually increased in size, and she felt discomfort in the right cheek area. The mass showed similar signal intensity to cerebrospinal fluid on T1-weighted images and T2-weighted images, but high signal intensity on fluid-attenuated inversion recovery images and diffusion-weighted images. Because the presence of very faint contrast enhancement was not noticed, the mass was preoperatively diagnosed as an epidermoid cyst. Tumor resection was performed, and the histopathological diagnosis was chondroid chordoma. Since intradural chordoma may resemble an epidermoid cyst on imaging, radiologists should check carefully for the presence of contrast enhancement and suggest the possibility of intradural chordoma.

## Introduction

Chordomas are rare, locally aggressive, primary bone tumors derived from primitive notochord remnants. In the skull base, they often arise from the spheno-occipital synchondrosis of the clivus. Although the vast majority of them show extradural extension and extensive bone destruction, chordomas that are located entirely within the intradural space have rarely been reported [1]. Furthermore, intradural chordomas with faint or absent contrast enhancement are even rarer and resemble imaging findings of epidermoid cysts. To the best of our knowledge, there have been four case reports of intradural chordomas mimicking epidermoid cysts on imaging [2-5]. Thus, the current study is the fifth case of an intradural chordoma in the prepontine cistern that mimicked an epidermoid cyst on imaging.

## Case presentation

**Figure 1:**
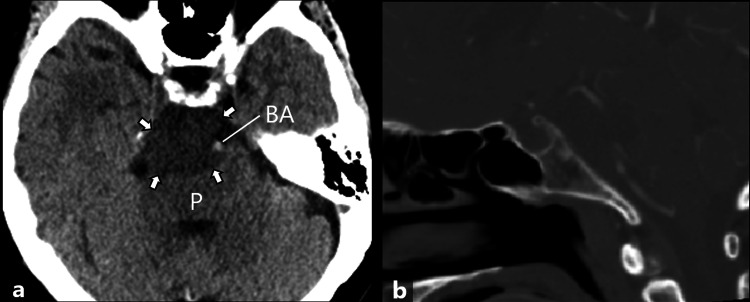
Noncontrast-enhanced head CT of the patient. The images show (a) axial noncontrast-enhanced CT and (b) sagittal image of the contrast-enhanced CT in the bone window. Noncontrast-enhanced head CT shows a 40-mm, well-defined, homogeneous, low-density mass in the prepontine cistern (a: arrows). The pons (P) and basilar artery (BA) are compressed dorsally by the mass. There is no obvious fat or calcification within the mass. No bone destruction of the clivus is seen (b).

A 72-year-old woman presented to our hospital due to right cheek area discomfort. She had a history of hypertension and dyslipidemia and was on medication. Twelve years earlier, she was incidentally found to have a mass in the prepontine cistern on magnetic resonance imaging (MRI). The mass was initially diagnosed as an epidermoid cyst and was followed up by MRI. On follow-up MRI, the mass gradually increased in size. At our hospital, a physical examination showed abnormal sensation in the second branch region of the right trigeminal nerve. Other physical examinations and laboratory findings were unremarkable. Noncontrast-enhanced head computed tomography (CT) showed a 40-mm, well-defined, homogeneous, low-density mass in the prepontine cistern (Figures 1a, 1b).

**Figure 2:**
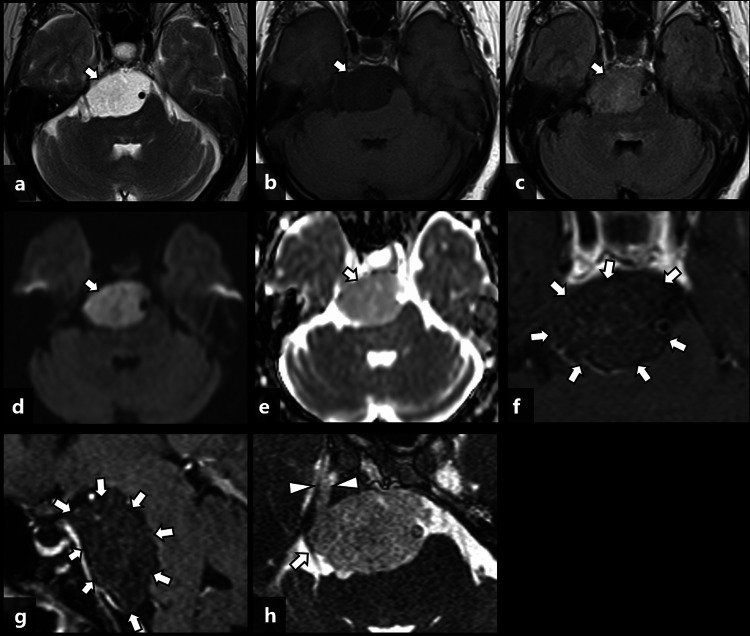
Brain MRI of the patient. The images show (a) axial T2-weighted image (T2WI), (b) axial T1-weighted image (T1WI), (c) axial fluid-attenuated inversion recovery (FLAIR) image, (d) axial diffusion-weighted image (DWI) (b: factor: 1000 s/mm^2^), (e) apparent diffusion coefficient (ADC) map, (f) axial contrast-enhanced T1WI, (g) sagittal contrast-enhanced fat-suppressed T1WI, and (h) axial reformatted image of constructive interference in steady state (CISS). The mass shows markedly high signal intensity on T2WI, similar to cerebrospinal fluid (CSF), and low signal intensity on T1WI (a, b: arrows) but mildly high signal intensity on FLAIR and DWI images (c, d: arrows). The ADC map shows lower signal intensity than CSF and higher signal intensity than brain parenchyma (e: arrow). Contrast-enhanced T1WI shows very faint honeycombed enhancement (f, g: arrows). The tumor extends into the right Meckel’s cave (h: arrowheads), and the right trigeminal nerve is compressed laterally (h: arrow).

The mass had no obvious fat or calcification. The midbrain, pons, and basilar artery were compressed dorsally by the mass. There was no obvious bone destruction of the clivus by the mass. On MRI, the mass showed low signal intensity on T1-weighted images (T1WI) and markedly high signal intensity on T2-weighted images (T2WI), which was similar to cerebrospinal fluid (CSF), but it showed mildly high signal intensity on fluid-attenuated inversion recovery (FLAIR) images (Figures 2a-2h).

Diffusion-weighted images (DWI) with a b-value of 1000 s/mm^2^ showed high signal intensity and apparent diffusion coefficient maps showed higher signal intensity than brain parenchyma and lower signal intensity than CSF (apparent diffusion coefficient {ADC} value: 1.13×10^-3^ mm^2^/s). Contrast-enhanced T1WI showed very faint honeycombed enhancement within the mass. Constructive interference in steady state (CISS) imaging showed that the mass extended into the right Meckel’s cave and compressed the right trigeminal nerve laterally. At this point, the presence of faint contrast enhancement within the mass was not noticed. As a result, the patient was diagnosed with an epidermoid cyst because of its similar signal intensity to CSF on T1WI and T2WI with high signal intensity on DWI.

**Figure 3:**
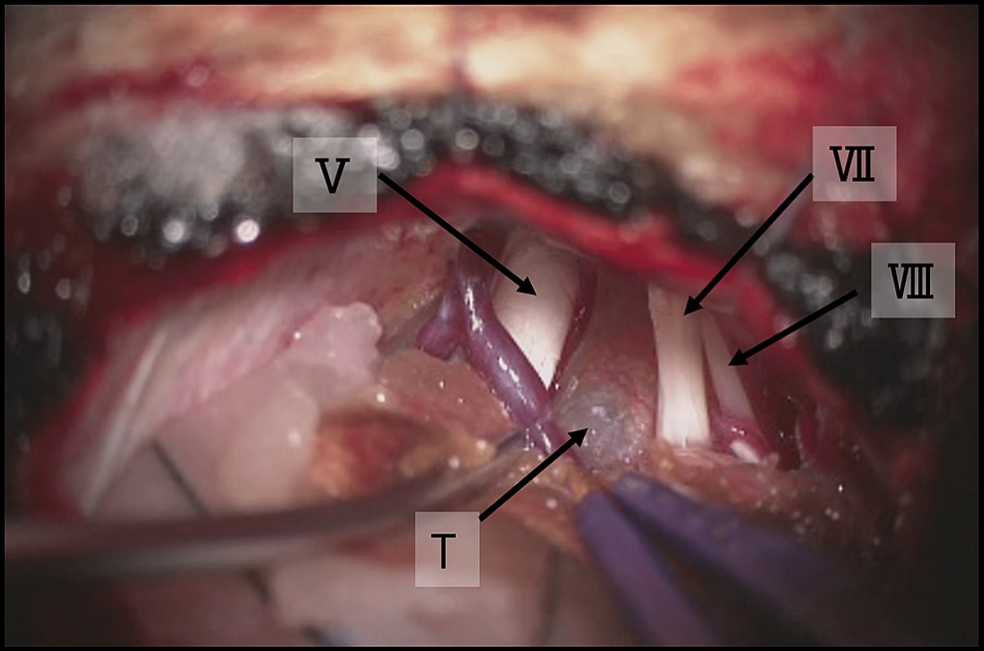
Intraoperative findings of the patient. View of the right cerebellar pontine angle observed from behind. A white, foamy tumor (T) is seen beyond the trigeminal (V), facial (VII), and auditory (VIII) nerves.

The patient underwent tumor resection because the mass had a tendency to increase in size and cause symptoms. Intraoperative findings showed that the tumor was completely located in the intradural space. The brainstem, the right oculomotor nerve, the right facial nerve, and the right vestibulocochlear nerve were compressed laterally by the mass (Figure 3).

**Figure 4:**
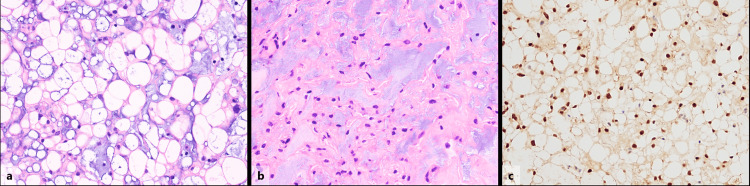
Histopathological findings of the tumor. The images show (a, b) hematoxylin-eosin staining and (c) brachyury staining. Tumor cells with vacuolated or pallid cytoplasm are proliferating in irregularly shaped cord-like or foci-like fashion (a) and are accompanied by myxoid or cartilage-like stroma (b). Immunohistological findings show positive staining for brachyury stain (c). These findings are consistent with chondroid chordoma.

Although almost complete tumor was resected, a very small amount of tumor remained near the basilar artery. A jelly-like tissue fragment was submitted for histopathology. Hematoxylin-eosin staining showed that tumor cells with vacuolated or pallid cytoplasm were proliferating in irregularly shaped cord-like or foci-like fashion, accompanied by myxoid or cartilage-like stroma (Figure 4a-4c).

Mild nuclear size discrepancy was observed, but nuclear fission was unremarkable. Immunohistological findings were positive for cytokeratin AE1/AE3, epithelial membrane antigen (EMA), S100, and brachyury. Based on these findings, the tumor was diagnosed as chondroid chordoma.

**Figure 5:**
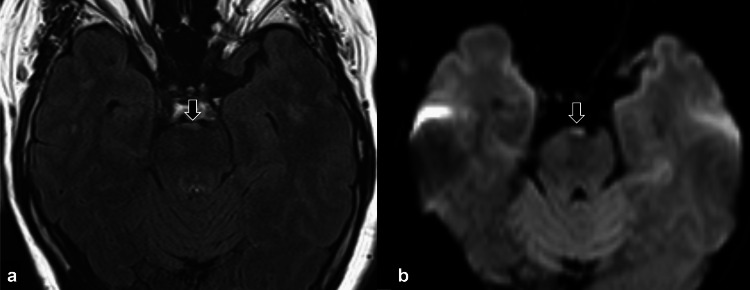
Postoperative MRI of the brain. The images show (a) axial fluid-attenuated inversion recovery (FLAIR) image and (b) axial diffusion-weighted image (DWI) (b factor: 1000 s/mm^2^). A very small amount of tumor remains near the basilar artery, which shows high signal intensity on FLAIR and DWI (a, b: arrows).

The right cheek area discomfort disappeared postoperatively, but the patient had right-sided hearing loss, mild restriction of right-sided ocular abduction, and mild truncal ataxia. Postoperative MRI showed a small amount of residual tumor near the basilar artery (Figures 5a, 5b). Two years after surgery, there was no residual tumor growth.

## Discussion

Chordomas are rare tumors arising from primitive notochord remnants, accounting for 1-4% of intracranial bone tumors. The sacrococcygeal region is the most commonly affected, at approximately 50%, followed by the skull base at 35% [6]. The prevalence is 0.08/100,000 persons, with a male-to-female ratio of approximately 2:1, and most cases occur in the 50s and 60s years of age [7]. Chordomas are histopathologically classified into four following types: conventional chordoma, chondroid chordoma, dedifferentiated chordoma, and poorly differentiated chordoma [8]. Chondroid chordoma is a subtype of conventional chordoma containing extracellular matrix mimicking hyaline cartilage.

Imaging plays a crucial role in the diagnosis and management of chordomas. On CT, chordomas usually present as a median-occurring, well-defined, hypoattenuating, osteolytic lesion with extensive bone destruction [9]. Intratumoral calcification (or bone fragments resulting from bone destruction) can be seen, which is considered more characteristic in chondroid chordoma than in conventional chordoma [10]. On MRI, chordomas generally show intermediate to low signal intensity on T1WI and markedly high signal intensity on T2WI. Most chordomas show moderate to marked contrast enhancement, but sometimes they show slight or absent enhancement, and rarely, honeycomb-like enhancement [11,12]. This may be due to necrosis or large amounts of mucinous material within the tumor. Reflecting mucin and cartilage matrix, the ADC values tend to be high (1.474±0.117×10^-3^ mm^2^/s), but poorly differentiated chordomas show lower signal intensity on T2WI and ADC values (0.875±0.100×10^-3^ mm^2^/s), reflecting the increased tumor cell density [13].

Although chordomas generally present as extradural, entirely intradural chordomas have rarely been reported. To date, approximately 47 cases of intradural chordoma have been reported, most of which were located within the prepontine cistern [1]. In addition, intradural chordomas originating in the parasellar region, foramen magnum occipitalis, hypothalamus, Meckel’s cave, pineal region, tentorium cerebelli, and cerebellopontine angle have been reported. The clinical features of intradural chordomas are less aggressive, their prognosis is better than that of typical chordomas, and most reported cases of intradural chordomas were free of recurrence [1]. However, although rare, cases of dissemination and delayed local recurrence have also been reported [14].

The pathogenesis of intradural chordoma is still unclear. Two hypotheses have been proposed to date. One proposed hypothesis is ectopic localization or migration of primitive notochord remnant. Three-dimensional reconstruction studies using serial sections of human embryos have shown that there are minute tissue projections and fragments called “migration forks” near the ventral skull base [15]. It has been proposed that these remnant fragments in the extradural compartment have the potential to migrate into the intradural space, including in the setting of trauma, and also lead to the development of intradural chordomas. Another hypothesis is malignant transformation of the ecchondrosis physaliphora (EP), an ectopic hyperostotic lesion of the notochordal remnant, usually located dorsal to the clivus via a cartilaginous or osseous stalk. An EP is usually less than 2 cm in size, asymptomatic, and found in 2% of autopsied cases [16]. It typically shows low signal intensity on T1WI and high signal intensity on T2WI [17]. The absence of contrast enhancement and an osseous stalk have been reported as differentiating features from chordoma on imaging [18].

In the present case, the intradural chordoma had been followed up as an epidermoid cyst because its faint honeycombed enhancement was not noticed. Epidermoid cysts occur most frequently in the cerebellopontine angle and typically show similar signal intensity to CSF on T1WI and T2WI without contrast enhancement and marked high signal intensity on DWI due to a combination of the T2-shine-through effect and true restricted diffusion [19]. The mean ADC value of epidermoid cysts was reported to be 1.197×10^-3^ mm^2^/s in a previous report, which was similar to that of the present case (1.13×10^-3^ mm^2^/s) [20]. In the present case, the presence of faint honeycombed contrast enhancement within the mass was thought to be more useful than the ADC value in differentiating an intradural chordoma from an epidermoid cyst. To date, there have been four reports of an intradural chordoma that mimicked an epidermoid cyst on imaging [2-5]. Table 1 summarizes the patients’ characteristics of reported and the present case.

**Table 1:** Summary of reported cases of intradural chordoma mimicking an epidermoid cyst. MRI: magnetic resonance imaging; T1WI: T1-weighted image; T2WI: T2-weighted image; FLAIR: fluid-attenuated inversion recovery; DWI: diffusion-weighted image; ADC: apparent diffusion coefficient; CE: contrast enhancement; NA: not available

Case	Author (reference)	Age (years)	Sex	Location	MRI findings							Histopathological findings
					Bone destruction	T1WI	T2WI	FLAIR	DWI	ADC map	CE	
1	Cho et al. [2]	32	M	Prepontine cistern	-	Low	High	NA	High	NA	Small reticulonodular enhancement	Conventional chordoma
2	de Almeida et al. [3]	44	M	Prepontine cistern	-	Low	High	Intermediate	High	NA	No enhancement	Conventional chordoma
3	Kim et al. [4]	27	M	Prepontine cistern	-	Low	High	Intermediate	High	Slightly low	No enhancement	Conventional chordoma
4	Ozek et al. [5]	35	F	Prepontine cistern	-	Low	High	NA	High	NA	No enhancement	Conventional chordoma
5	Present case	72	F	Prepontine cistern	-	Low	High	Intermediate	High	Slightly low	Faint honeycombed enhancement	Chondroid chordoma

Of the four reported cases, faint contrast enhancement was reported in one case, and the remaining three cases were reported to have no contrast enhancement. However, we reviewed the available images of the remaining three cases and found very faint contrast enhancement in two cases. In total, contrast enhancement was found in three of the four reported cases. None of the four reported cases was diagnosed with intradural chordoma preoperatively, as in the present case. Careful observation of the presence of contrast enhancement could have helped diagnose intradural chordoma preoperatively.

## Conclusions

In conclusion, a case of an intradural chordoma that mimicked an epidermoid cyst on imaging was described. Chordomas usually present as extradural tumors, but rarely they present as entirely intradural tumors and may resemble an epidermoid cyst on imaging. Therefore, radiologists should check carefully for the presence of contrast enhancement and suggest the possibility of intradural chordoma.
